# Food science articles in a post-COVID-19 era

**DOI:** 10.1007/s44187-021-00003-3

**Published:** 2021-12-16

**Authors:** Charis M. Galanakis

**Affiliations:** 1 Research and Innovation Department, Galanakis Laboratories, Chania, Greece; 2 Food Waste Recovery Group, ISEKI Food Association, Vienna, Austria; 3 Taif University, Taif, Saudi Arabia

The outbreak of the COVID-19 pandemic in early 2020 caused an emerging health crisis and repeated lockdowns that restricted human activities, causing a sudden physical and psychological disruption to citizens, markets, and societal and economic sub-systems. As proved in practice, public authorities, businesses, and citizens were unprepared for this rare crisis that impacted governmental policies and societies and led to a worldwide recession. The food sector was not an exception, and thus today the transition of the food industry to the post-pandemic period is imperative. As the world has placed hope on vaccination progress to recover the daily life and what we perceived as “normality” 2 years ago, the pandemic has changed the way we think, impacting consumers’ behavior and accelerating pre-existing trends.

## The pandemic has shifted the food sector towards safety, bioactivity, security, resilience, and sustainability

In the post-pandemic era, the research in the food sector was abruptly redirected to four main domains [3]. Firstly, food safety became a critical issue to limit the spreading of the virus in the food chain among producers, farmers, retailers, and consumers [7]. Secondly, the demand for foods containing bioactive compounds increased rapidly, as consumers were looking to adopt healthier diets and boost their immune systems [5]. Thirdly, the lockdown of billions of people worldwide has led to food shortages and food security problems related to price spikes and panic buying [2]. Finally, the sustainability and resilience of the food sector is another issue to be addressed to restrict relevant crises in the future [1].

Following these advances, emerging technologies to improve the functionality of food ingredients [4] together with internet and communication technologies (ICTs), Industry 4.0, and blockchain applications in the food supply chain are the innovations with the highest potential in the new era [6]. There is also hype for new approaches and investigations that redefine our food consumption norms, such as plant-based protein alternatives, artificial meat, and the valorization of food processing by-products. In addition, we need to understand the perceptions and attitudes of modern consumers and identify relevant barriers. These new directions open doors for green innovations and technology disruptions in the food sector within the lockdown and post-lockdown periods but also redefine the role of the food sector as a part of the bioeconomy and climate neutral economy in the years to come [6, 8].

## The pandemic has changed the scientific publishing industry towards accessibility and review speed

Research is widely presented and evaluated through peer-reviewed articles. Other communication tools (e.g., conference presentations, chapters in books, teaching modules, social media accounts, videos, etc.) are vital for research. However, the publication of articles in scientific journals remains the essential means of communication, since the submitted manuscripts should typically include some key elements: an introduction, the state-of-the-field, the progress upon it, and the scope of the study, a valid research methodology, presentation of results and justified outcomes in the discussion. These elements are evaluated through the peer-review process of scientific journals.

On the other hand, the pandemic has also impacted the publishing industry and modified it forever. Since there was limited knowledge about SARS-CoV-2 and COVID-19 disease at the beginning of the crisis, the urgent need for fast-track prevention and treatment and re-arrangement of societal strategies led researchers to publish articles in “torrents”: high-speed and accessible to all via posts on websites and blogs. This practice has a main disadvantage: the noted research had not been evaluated and peer-reviewed by experts in the field. Nevertheless, since the information and knowledge are today spread extremely fast, and simultaneously the researchers’ pool is increasing exponentially worldwide, an urgent need for fast and reliable research outcomes has been generated. This need should be covered with means that are openly accessible to the public, but at the same time, are sustainable and not too expensive for the majority of the researchers. For example, many funding agencies require researchers to publish only in open access (OA) journals.

In addition, the practice of submitting a manuscript to a journal and then waiting for three, four, or more months to get a response belongs to the previous century. For instance, last year, we submitted a review article to a high-impact journal and received reviewers’ reports after 7–8 months. This waiting period is so long that some of the reviewers’ criticism and comments concerned knowledge published after the initial submission date. The new generation of researchers, millennials, cannot wait that long. Instead, flexibility and fast and accurate decision-making are the critical aspects of success for modern researchers. This is not a rough and sketchy approach; it is an evolution for the publishing industry based on improved handling, and a realistic strategy for all the journals in the new era.

All researchers read many papers daily, so a couple of weeks for the review process and less than a month for the editor's decision are reasonable and realistic timeframes for all journals. Subsequently, publishers should adapt to the new era by switching some of their journals to OA or providing this kind of possibility to researchers at a reasonable publishing cost and supporting researchers to communicate their work related to the world's most critical challenges.

## Discover food in the new era

Considering these trends, Discover Food is a transdisciplinary and OA journal that aspires to fill the gap in the food science publications sector. It provides an innovative platform for the rapid dissemination of knowledge included in high-impact research articles, original papers, reviews, viewpoints, case studies, and short communications covering the whole spectrum of research conducted for the food sector. The journal welcomes basic, methodological, and applied science that focuses on food science, technology, and nutrition, and advances are taken at all stages of the food supply chain. It will also publish papers that deal with future transitions of the food sector towards a climate-neutral economy and sustainable development. Emphasis will be given to studies that contribute to the United Nations Sustainable Development Goals (SDGs) and specifically those addressing SDG2 (zero hunger), SDG3 (good health and well-being), SDG 12 (responsible consumption and production), SDG14 (life below water) and SDG 15 (life on land). Topics of urgent interest are food waste recovery, and reutilization of target compounds in the food chain, the sustainability of food systems and saving food efforts, innovations strategies, biobased industries, non-thermal processing, food quality and shelf-life, innovations in food analysis, and traditional foods, food authentication, and food toxicology, and functional food ingredients such as herbs and additives, but also essential food components like polyphenols, glucosinolates, carotenoids, lipids, proteins and peptides, and dietary fiber. The COVID-19 food systems require disruptive technologies, enhanced system resilience, green recovery, food safety, transparency from farm to fork, and food products towards personalized nutrition. Welcome to Discover Food!
